# Supplementary material for: Evolutionarily new genes in humans with disease phenotypes reveal functional enrichment patterns shaped by adaptive innovation and sexual selection
Source: Genome Res. 2025 Mar;35(3):379–92. doi: 10.1101/gr.279498.124 (PMC11960464; doi:10.1101/gr.279498.124)
Supplement: Supplement 14 [file Supplemental_Material.docx]

**Supplemental Material**

**The general pattern involving gene age and Mendelian diseases**

After the discovery of the first disease gene in 1983, which was based on linkage mapping for a Huntington's disease with a pedigree (Gusella et al. 1983), there has been a rapid advancement in medical genetics research. As of now, this field has identified approximately 20% of human genes (~4000-5000 genes) associated with rare diseases, "orphan" diseases, and rare forms of common diseases (Asimit et al. 2012; Boycott et al. 2013; Henn et al. 2015; Krumm et al. 2015; Ji et al. 2016; Study 2017; Krausz and Riera-Escamilla 2018; Wen et al. 2018; Almlöf et al. 2019; Povysil et al. 2019; Thuresson et al. 2019; Guo et al. 2021; Oud et al. 2021; Chen et al. 2022). In our study, we utilized the latest disease gene and clinical phenotype data from HPO annotations (Köhler et al. 2018) and incorporated synteny-based gene age dating to account for new gene duplication events (Shao et al. 2019). Our synteny-based gene age dating reveals that younger genes have lower percentages of disease genes than older genes, qualitatively consistent with previous findings (Domazet-Lošo and Tautz 2008). We also reveal that evolutionary older genes tend to have a higher gene-wise DNVs burden. Logistic regression modeling indicates that protein length, gene age, and DNV burden are positively correlated with the probability of a gene being classified as a disease gene. Thus, the overrepresentation of disease genes in older evolutionary age groups could result from the combined effect of deleterious variant burden, sequence length, and gene age over evolutionary time under various forms of selection. Despite previous debates on the selective pressures on disease genes (Smith and Eyre-Walker 2003; Domazet-Lošo and Tautz 2008; Chakraborty et al. 2016; Spataro et al. 2017), *K*_a_/*K*_s_ ratios between humans and primates consistently support stronger purifying selection on disease genes than non-disease genes, indicating evolutionary constraints to remove harmful variants. The phylostratum-wise estimates of the emergence rate of disease genes per million years reveal a steady integration of new genes into disease phenotypes, consistent with Haldane's finding that new deleterious mutations are eliminated at the same rate they occur (Haldane 1937; Keightley 2012).

The excess of disease genes in the X Chromosome supports the “faster-X effect” (Rice 1984; Charlesworth et al. 1987), that male X-hemizygosity could immediately expose the deleterious X chromosomal mutations to purifying selection. Conversely, the X-Chromosome inactivation (XCI) in female cells could alleviate the deleterious phenotypes of disease variants on the X Chromosome (Migeon 2020). The X Chromosome excess of disease genes is attributed disproportionally to genes with male reproductive functions. This male-specific bias is not limited to the sex chromosome but also detectable in autosomes. These findings align with the “faster-male” effect, where the reproductive system evolves more rapidly in males than in females due to heightened male-specific sexual selection (Wu and Davis 1993). Of the 22 HPO systems, young genes are enriched in disease phenotypes affecting the reproductive-related system. As genes evolve to be older, there's a marked decline in both phenotype enrichment and the male-to-female ratio of reproductive disease gene numbers, consistent with the “out of testis” hypothesis (Kaessmann 2010). This hypothesis also predicts that new genes could gain broader expression patterns and higher phenotypic complexity over evolutionary time (Vinckenbosch et al. 2006). Consistently, we reveal a pattern where older sets of disease genes have phenotypes affecting a much broader range of anatomical systems compared to younger genes. The strong enrichment of male reproductive phenotypes for young genes is also consistent with findings from model species that new genes often exhibit male-reproductive expression and functions (Betrán et al. 2002; Heinen et al. 2009), in both *Drosophila* (Heinen et al. 2009; Gubala et al. 2017; VanKuren and Long 2018) and mammals (Emerson et al. 2004; Jiang et al. 2017). Some new gene duplicates on autosomes are indispensable during male spermatogenesis, to preserve male-specific functions that would otherwise be silenced on the X Chromosome due to the meiotic sex chromosome inactivation (MSCI) (Emerson et al. 2004; Zhang et al. 2010; Jiang et al. 2017).

**Evolutionary new genes are frequent source of adaptive innovation**

Apart from the reproductive functions, new genes are also enriched for adaptive phenotypes. Previous transcriptomic studies indicate that new genes have excessive upregulation in the human neocortex under positive selection (Zhang et al. 2011). The brain size enlargement ranks among the most extraordinary human phenotypic innovations (Rakic 2009; Zhang et al. 2011). Here, we found that a high proportion of primate-specific disease genes (42%) affecting the nervous systems could impact phenotypes related to brain size and intellectual development. For example, *DDX11* is critical in pathology of microcephaly (Pirozzi et al. 2018; Lerner et al. 2020; van Schie et al. 2020; Ma et al. 2022). The *NOTCH2NLA*, *NOTCH2NLB*, and *NOTCH2NLC* may promote human brain size enlargement, due to their functions in neuronal intranuclear inclusion disease (NIID), microcephaly, and macrocephaly (Fiddes et al. 2018; Suzuki et al. 2018; Liu et al. 2022). The *RRP7A* is also a microcephaly disease gene evidenced from patient-derived cells with defects in cell cycle progression and primary cilia resorption (Farooq et al. 2020). The defects of *SMPD4* can lead to a neurodevelopmental disorder characterized by microcephaly and structural brain anomalies (Magini et al. 2019). The *SRGAP2C* accounts for human-specific feature of neoteny and can promote motor and execution skills in mouse and monkey model (Charrier et al. 2012; Dennis et al. 2012; Meng et al. 2023).

Young genes are also enriched in other adaptive phenotypes, such as the head and neck, eyes, and musculoskeletal system. Some examples include *CFHR3* associated with macular degeneration (Fritsche et al. 2016), *SMPD4* with the retinopathy (Smits et al. 2023), *TUBA3D* with the keratoconus (Hao et al. 2017), *OPN1MW* with loss of color vision (Winderickx et al. 1992; Ueyama et al. 2002), *YY1AP1* with Fibromuscular dysplasia (Guo et al. 2017), *SMN2* with spinal muscular atrophy (Hahnen et al. 1996), *GH1* with defects in adult bone mass and bone loss (Dennison et al. 2004), *KCNJ18* with thyrotoxicosis complicated by paraplegia and hyporeflexia (Ryan et al. 2010), *TBX5* with the cardiac and limb defects of Holt-Oram syndrome (Basson et al. 1997; Li et al. 1997), and *DUX4* with muscular dystrophy (Lemmers et al. 2012). Additionally, sex-specific functions have also been reported for these young genes. For example, the Y chromosome gene *TBL1Y* could lead to male-specific hearing loss (Di Stazio et al. 2019). Defects in *TUBB8* could lead to complete cleavage failure in fertilized eggs and oocyte maturation arrest (Feng et al. 2016; Yuan et al. 2018; Yao et al. 2022). Interestingly, a previous case study on mice also shows the role of de novo genes in female-specific reproductive functions (Xie et al. 2019).

**Reference**

Almlöf JC, Nystedt S, Leonard D, Eloranta M-L, Grosso G, Sjöwall C, Bengtsson AA, Jönsen A, Gunnarsson I, Svenungsson E. 2019. Whole-genome sequencing identifies complex contributions to genetic risk by variants in genes causing monogenic systemic lupus erythematosus. *Human genetics* **138**: 141-150.

Asimit JL, Day-Williams AG, Morris AP, Zeggini E. 2012. ARIEL and AMELIA: testing for an accumulation of rare variants using next-generation sequencing data. *Human heredity* **73**: 84-94.

Basson CT, Bachinsky DR, Lin RC, Levi T, Elkins JA, Soults J, Grayzel D, Kroumpouzou E, Traill TA, Leblanc-Straceski J et al. 1997. Mutations in human TBX5 [corrected] cause limb and cardiac malformation in Holt-Oram syndrome. *Nat Genet* **15**: 30-35.

Betrán E, Thornton K, Long M. 2002. Retroposed New Genes Out of the X in Drosophila. *Genome Research* **12**: 1854-1859.

Boycott KM, Vanstone MR, Bulman DE, MacKenzie AE. 2013. Rare-disease genetics in the era of next-generation sequencing: discovery to translation. *Nature Reviews Genetics* **14**: 681-691.

Chakraborty S, Panda A, Ghosh TC. 2016. Exploring the evolutionary rate differences between human disease and non-disease genes. *Genomics* **108**: 18-24.

Charlesworth B, Coyne JA, Barton NH. 1987. The relative rates of evolution of sex chromosomes and autosomes. *The American Naturalist* **130**: 113-146.

Charrier C, Joshi K, Coutinho-Budd J, Kim J-E, Lambert N, De Marchena J, Jin W-L, Vanderhaeghen P, Ghosh A, Sassa T. 2012. Inhibition of SRGAP2 function by its human-specific paralogs induces neoteny during spine maturation. *Cell* **149**: 923-935.

Chen J, Zhang P, Chen H, Wang X, He X, Zhong J, Zheng H, Li X, Jakovlić I, Zhang Y et al. 2022. Whole-genome sequencing identifies rare missense variants of WNT16 and ERVW-1 causing the systemic lupus erythematosus. *Genomics* **114**: 110332.

Dennis MY, Nuttle X, Sudmant PH, Antonacci F, Graves TA, Nefedov M, Rosenfeld JA, Sajjadian S, Malig M, Kotkiewicz H. 2012. Evolution of human-specific neural SRGAP2 genes by incomplete segmental duplication. *Cell* **149**: 912-922.

Dennison EM, Syddall HE, Rodriguez S, Voropanov A, Day IN, Cooper C. 2004. Polymorphism in the growth hormone gene, weight in infancy, and adult bone mass. *J Clin Endocrinol Metab* **89**: 4898-4903.

Di Stazio M, Collesi C, Vozzi D, Liu W, Myers M, Morgan A, D′Adamo PA, Girotto G, Rubinato E, Giacca M et al. 2019. TBL1Y: a new gene involved in syndromic hearing loss. *European Journal of Human Genetics* **27**: 466-474.

Domazet-Lošo T, Tautz D. 2008. An ancient evolutionary origin of genes associated with human genetic diseases. *Molecular biology and evolution* **25**: 2699-2707.

Emerson J, Kaessmann H, Betrán E, Long M. 2004. Extensive gene traffic on the mammalian X chromosome. *Science* **303**: 537-540.

Farooq M, Lindbæk L, Krogh N, Doganli C, Keller C, Mönnich M, Gonçalves AB, Sakthivel S, Mang Y, Fatima A et al. 2020. RRP7A links primary microcephaly to dysfunction of ribosome biogenesis, resorption of primary cilia, and neurogenesis. *Nat Commun* **11**: 5816.

Feng R, Sang Q, Kuang Y, Sun X, Yan Z, Zhang S, Shi J, Tian G, Luchniak A, Fukuda Y et al. 2016. Mutations in TUBB8 and Human Oocyte Meiotic Arrest. *New England Journal of Medicine* **374**: 223-232.

Fiddes IT, Lodewijk GA, Meghan M, Bosworth CM, Ewing AD, Mantalas GL, Novak AM, Anouk VDB, Alex B, Rosenkrantz JL. 2018. Human-Specific NOTCH2NL Genes Affect Notch Signaling and Cortical Neurogenesis. *Cell* **173**: 1356-1369.e1322.

Fritsche LG Igl W Bailey JNC Grassmann F Sengupta S Bragg-Gresham JL Burdon KP Hebbring SJ Wen C Gorski M et al. 2016. A large genome-wide association study of age-related macular degeneration highlights contributions of rare and common variants. *Nature genetics* **48**: 134-143.

Gubala AM, Schmitz JF, Kearns MJ, Vinh TT, Bornberg-Bauer E, Wolfner MF, Findlay GD. 2017. The goddard and saturn genes are essential for Drosophila male fertility and may have arisen de novo. *Molecular biology and evolution* **34**: 1066-1082.

Guo DC, Duan XY, Regalado ES, Mellor-Crummey L, Kwartler CS, Kim D, Lieberman K, de Vries BBA, Pfundt R, Schinzel A et al. 2017. Loss-of-Function Mutations in YY1AP1 Lead to Grange Syndrome and a Fibromuscular Dysplasia-Like Vascular Disease. *Am J Hum Genet* **100**: 21-30.

Guo T, Tu C-F, Yang D-H, Ding S-Z, Lei C, Wang R-C, Liu L, Kang X, Shen X-Q, Yang Y-F et al. 2021. Bi-allelic BRWD1 variants cause male infertility with asthenoteratozoospermia and likely primary ciliary dyskinesia. *Human Genetics* **140**: 761-773.

Gusella JF, Wexler NS, Conneally PM, Naylor SL, Anderson MA, Tanzi RE, Watkins PC, Ottina K, Wallace MR, Sakaguchi AY et al. 1983. A polymorphic DNA marker genetically linked to Huntington's disease. *Nature* **306**: 234-238.

Hahnen E, Schönling J, Rudnik-Schöneborn S, Zerres K, Wirth B. 1996. Hybrid survival motor neuron genes in patients with autosomal recessive spinal muscular atrophy: new insights into molecular mechanisms responsible for the disease. *Am J Hum Genet* **59**: 1057-1065.

Haldane J. 1937. The effect of variation of fitness. *The American Naturalist* **71**: 337-349.

Hao XD, Chen P, Zhang YY, Li SX, Shi WY, Gao H. 2017. De novo mutations of TUBA3D are associated with keratoconus. *Sci Rep* **7**: 13570.

Heinen TJ, Staubach F, Häming D, Tautz D. 2009. Emergence of a new gene from an intergenic region. *Current biology* **19**: 1527-1531.

Henn BM, Botigué LR, Bustamante CD, Clark AG, Gravel S. 2015. Estimating the mutation load in human genomes. *Nature Reviews Genetics* **16**: 333-343.

Ji X, Kember RL, Brown CD, Bućan M. 2016. Increased burden of deleterious variants in essential genes in autism spectrum disorder. *Proceedings of the National Academy of Sciences* **113**: 15054-15059.

Jiang L, Li T, Zhang X, Zhang B, Yu C, Li Y, Fan S, Jiang X, Khan T, Hao Q. 2017. RPL10L is required for male meiotic division by compensating for RPL10 during meiotic sex chromosome inactivation in mice. *Current Biology* **27**: 1498-1505. e1496.

Kaessmann H. 2010. Origins, evolution, and phenotypic impact of new genes. *Genome research* **20**: 1313-1326.

Keightley PD. 2012. Rates and fitness consequences of new mutations in humans. *Genetics* **190**: 295-304.

Köhler S, Carmody L, Vasilevsky N, Jacobsen JO B, Danis D, Gourdine J-P, Gargano M, Harris NL, Matentzoglu N, McMurry JA et al. 2018. Expansion of the Human Phenotype Ontology (HPO) knowledge base and resources. *Nucleic Acids Research* **47**: D1018-D1027.

Krausz C, Riera-Escamilla A. 2018. Genetics of male infertility. *Nature Reviews Urology* **15**: 369-384.

Krumm N, Turner TN, Baker C, Vives L, Mohajeri K, Witherspoon K, Raja A, Coe BP, Stessman HA, He Z-X et al. 2015. Excess of rare, inherited truncating mutations in autism. *Nature Genetics* **47**: 582-588.

Lemmers RJ, Tawil R, Petek LM, Balog J, Block GJ, Santen GW, Amell AM, van der Vliet PJ, Almomani R, Straasheijm KR et al. 2012. Digenic inheritance of an SMCHD1 mutation and an FSHD-permissive D4Z4 allele causes facioscapulohumeral muscular dystrophy type 2. *Nat Genet* **44**: 1370-1374.

Lerner LK, Holzer S, Kilkenny ML, Šviković S, Murat P, Schiavone D, Eldridge CB, Bittleston A, Maman JD, Branzei D et al. 2020. Timeless couples G-quadruplex detection with processing by DDX11 helicase during DNA replication. *The EMBO Journal* **39**: e104185.

Li QY, Newbury-Ecob RA, Terrett JA, Wilson DI, Curtis AR, Yi CH, Gebuhr T, Bullen PJ, Robson SC, Strachan T et al. 1997. Holt-Oram syndrome is caused by mutations in TBX5, a member of the Brachyury (T) gene family. *Nat Genet* **15**: 21-29.

Liu Q, Zhang K, Kang Y, Li Y, Deng P, Li Y, Tian Y, Sun Q, Tang Y, Xu K et al. 2022. Expression of expanded GGC repeats within NOTCH2NLC causes behavioral deficits and neurodegeneration in a mouse model of neuronal intranuclear inclusion disease. *Science Advances* **8**: eadd6391.

Ma C, Li C, Ma H, Yu D, Zhang Y, Zhang D, Su T, Wu J, Wang X, Zhang L et al. 2022. Pan-cancer surveys indicate cell cycle-related roles of primate-specific genes in tumors and embryonic cerebrum. *Genome Biology* **23**: 251.

Magini P, Smits DJ, Vandervore L, Schot R, Columbaro M, Kasteleijn E, van der Ent M, Palombo F, Lequin MH, Dremmen M et al. 2019. Loss of SMPD4 Causes a Developmental Disorder Characterized by Microcephaly and Congenital Arthrogryposis. *Am J Hum Genet* **105**: 689-705.

Meng X, Lin Q, Zeng X, Jiang J, Li M, Luo X, Chen K, Wu H, Hu Y, Liu C et al. 2023. Brain developmental and cortical connectivity changes in the transgenic monkeys carrying the human-specific duplicated gene srGAP2C. *National Science Review* doi:10.1093/nsr/nwad281.

Migeon BR. 2020. X-linked diseases: susceptible females. *Genetics in Medicine* **22**: 1156-1174.

Neme R, Tautz D. 2013. Phylogenetic patterns of emergence of new genes support a model of frequent de novoevolution. *BMC Genomics* **14**: 117.

Oud MS, Houston BJ, Volozonoka L, Mastrorosa FK, Holt GS, Alobaidi BKS, deVries PF, Astuti G, Ramos L, Mclachlan RI et al. 2021. Exome sequencing reveals variants in known and novel candidate genes for severe sperm motility disorders. *Human Reproduction* **36**: 2597-2611.

Pirozzi F, Nelson B, Mirzaa G. 2018. From microcephaly to megalencephaly: determinants of brain size. *Dialogues Clin Neurosci* **20**: 267-282.

Povysil G, Petrovski S, Hostyk J, Aggarwal V, Allen AS, Goldstein DB. 2019. Rare-variant collapsing analyses for complex traits: guidelines and applications. *Nature Reviews Genetics* **20**: 747-759.

Rakic P. 2009. Evolution of the neocortex: a perspective from developmental biology. *Nat Rev Neurosci* **10**: 724-735.

Rice WR. 1984. Sex chromosomes and the evolution of sexual dimorphism. *Evolution*: 735-742.

Ryan DP, da Silva MR, Soong TW, Fontaine B, Donaldson MR, Kung AW, Jongjaroenprasert W, Liang MC, Khoo DH, Cheah JS et al. 2010. Mutations in potassium channel Kir2.6 cause susceptibility to thyrotoxic hypokalemic periodic paralysis. *Cell* **140**: 88-98.

Shao Y, Chen C, Shen H, He BZ, Yu D, Jiang S, Zhao S, Gao Z, Zhu Z, Chen X. 2019. GenTree, an integrated resource for analyzing the evolution and function of primate-specific coding genes. *Genome research*.

Smith NG, Eyre-Walker A. 2003. Human disease genes: patterns and predictions. *Gene* **318**: 169-175.

Smits DJ, Schot R, Krusy N, Wiegmann K, Utermöhlen O, Mulder MT, den Hoedt S, Yoon G, Deshwar AR, Kresge C et al. 2023. SMPD4 regulates mitotic nuclear envelope dynamics and its loss causes microcephaly and diabetes. *Brain* **146**: 3528-3541.

Spataro N, Rodríguez JA, Navarro A, Bosch E. 2017. Properties of human disease genes and the role of genes linked to Mendelian disorders in complex disease aetiology. *Human molecular genetics* **26**: 489-500.

Study TDDD. 2017. Prevalence and architecture of de novo mutations in developmental disorders. *Nature* **542**: 433.

Suzuki IK, Gacquer D, Van Heurck R, Kumar D, Wojno M, Bilheu A, Herpoel A, Lambert N, Cheron J, Polleux F. 2018. Human-specific NOTCH2NL genes expand cortical neurogenesis through Delta/Notch regulation. *Cell* **173**: 1370-1384. e1316.

Thuresson AC, Zander CS, Zhao JJ, Halvardson J, Maqbool K, Månsson E, Stenninger E, Holmlund U, Öhrner Y, Feuk L. 2019. Whole genome sequencing of consanguineous families reveals novel pathogenic variants in intellectual disability. *Clinical genetics* **95**: 436.

Ueyama H, Kuwayama S, Imai H, Tanabe S, Oda S, Nishida Y, Wada A, Shichida Y, Yamade S. 2002. Novel missense mutations in red/green opsin genes in congenital color-vision deficiencies. *Biochem Biophys Res Commun* **294**: 205-209.

van Schie JJM, Faramarz A, Balk JA, Stewart GS, Cantelli E, Oostra AB, Rooimans MA, Parish JL, de Almeida Estéves C, Dumic K et al. 2020. Warsaw Breakage Syndrome associated DDX11 helicase resolves G-quadruplex structures to support sister chromatid cohesion. *Nature Communications* **11**: 4287.

VanKuren NW, Long M. 2018. Gene duplicates resolving sexual conflict rapidly evolved essential gametogenesis functions. *Nature ecology & evolution* **2**: 705-712.

Vinckenbosch N, Dupanloup I, Kaessmann H. 2006. Evolutionary fate of retroposed gene copies in the human genome. *Proceedings of the National Academy of Sciences* **103**: 3220-3225.

Wen L, Zhu C, Zhu Z, Yang C, Zheng X, Liu L, Zuo X, Sheng Y, Tang H, Liang B. 2018. Exome-wide association study identifies four novel loci for systemic lupus erythematosus in Han Chinese population. *Annals of the rheumatic diseases* **77**: 417-417.

Winderickx J, Sanocki E, Lindsey DT, Teller DY, Motulsky AG, Deeb SS. 1992. Defective colour vision associated with a missense mutation in the human green visual pigment gene. *Nature Genetics* **1**: 251-256.

Wu C-I, Davis AW. 1993. Evolution of postmating reproductive isolation: the composite nature of Haldane's rule and its genetic bases. *The American Naturalist* **142**: 187-212.

Xie C, Bekpen C, Künzel S, Keshavarz M, Krebs-Wheaton R, Skrabar N, Ullrich KK, Tautz D. 2019. A de novo evolved gene in the house mouse regulates female pregnancy cycles. *eLife* **8**: e44392.

Yao Z, Zeng J, Zhu H, Zhao J, Wang X, Xia Q, Li Y, Wu L. 2022. Mutation analysis of the TUBB8 gene in primary infertile women with oocyte maturation arrest. *Journal of Ovarian Research* **15**: 38.

Yuan P, Zheng L, Liang H, Li Y, Zhao H, Li R, Lai L, Zhang Q, Wang W. 2018. A novel mutation in the TUBB8 gene is associated with complete cleavage failure in fertilized eggs. *J Assist Reprod Genet* **35**: 1349-1356.

Zhang YE, Landback P, Vibranovski MD, Long M. 2011. Accelerated Recruitment of New Brain Development Genes into the Human Genome. *PLOS Biology* **9**: e1001179.

Zhang YE, Vibranovski MD, Landback P, Marais GA, Long M. 2010. Chromosomal redistribution of male-biased genes in mammalian evolution with two bursts of gene gain on the X chromosome. *PLoS biology* **8**: e1000494.

Zhao G, Li K, Li B, Wang Z, Fang Z, Wang X, Zhang Y, Luo T, Zhou Q, Wang L et al. 2020. Gene4Denovo: an integrated database and analytic platform for de novo mutations in humans. *Nucleic Acids Res* **48**: D913-d926.

Supplemental_Fig_S1


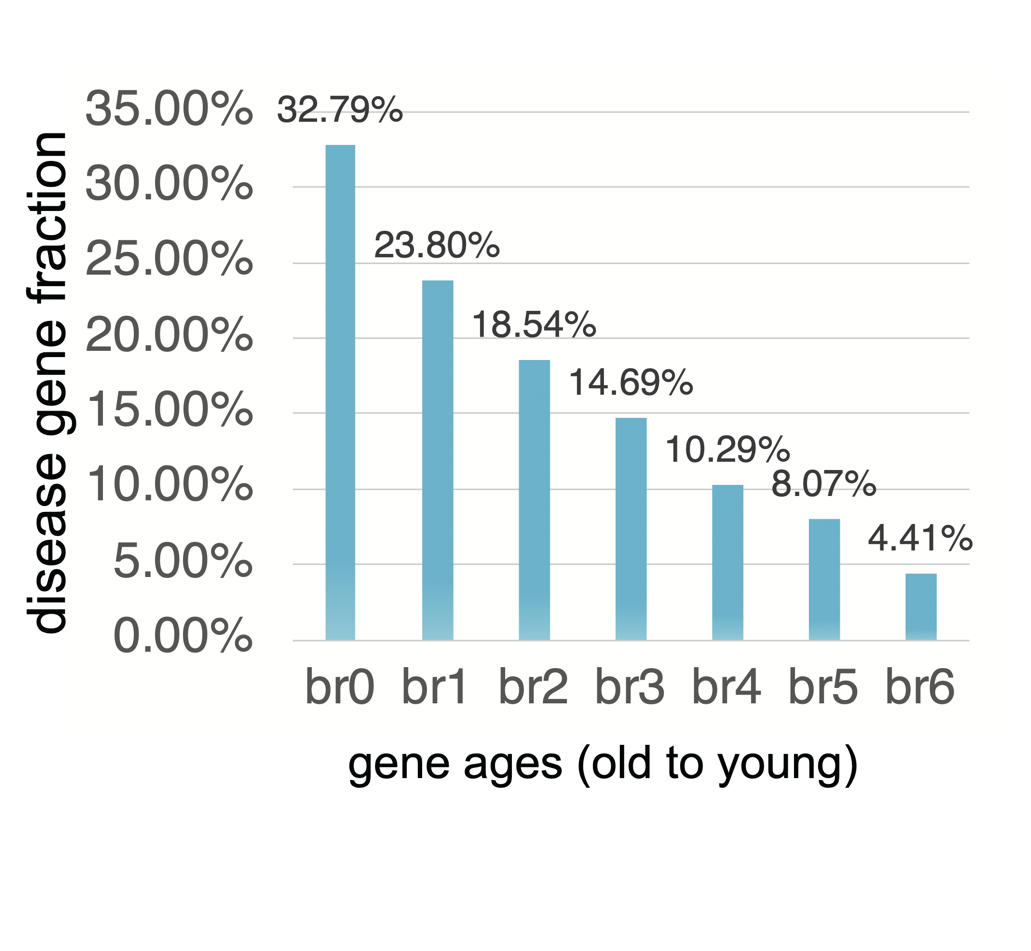


The fractions of disease genes for seven age groups (phylostrata). The horizontal axis shows the seven age groups and the vertical one shows the fractions of disease genes out of each age group. The “br” indicates “branch”, which is also age group or phylostratum.

Supplemental_Fig_S2
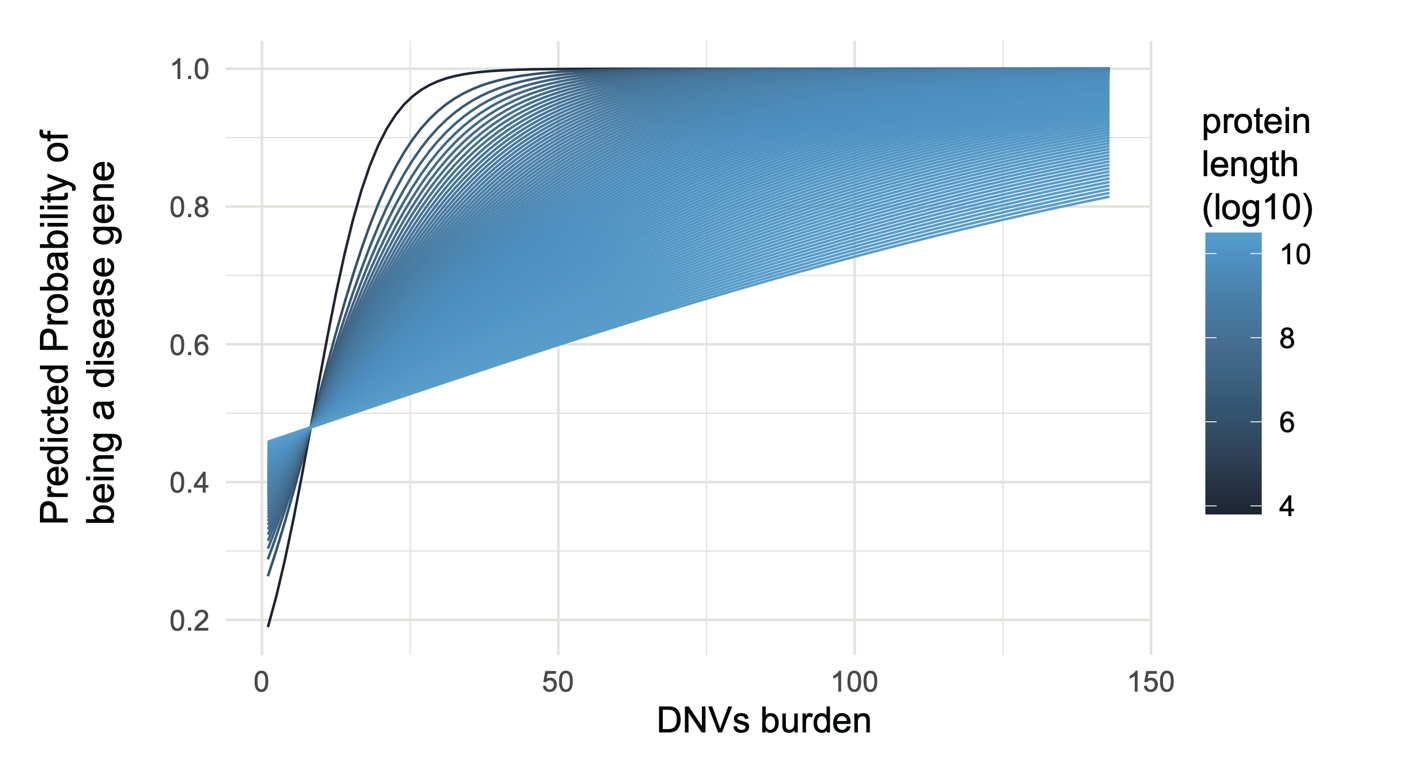


The interaction plot between DNVs burden and protein length (at logarithm scale) for the predicted probability of being a disease gene. The details of full model are shown in Supplemental Table 4.

Supplemental_Fig_S3


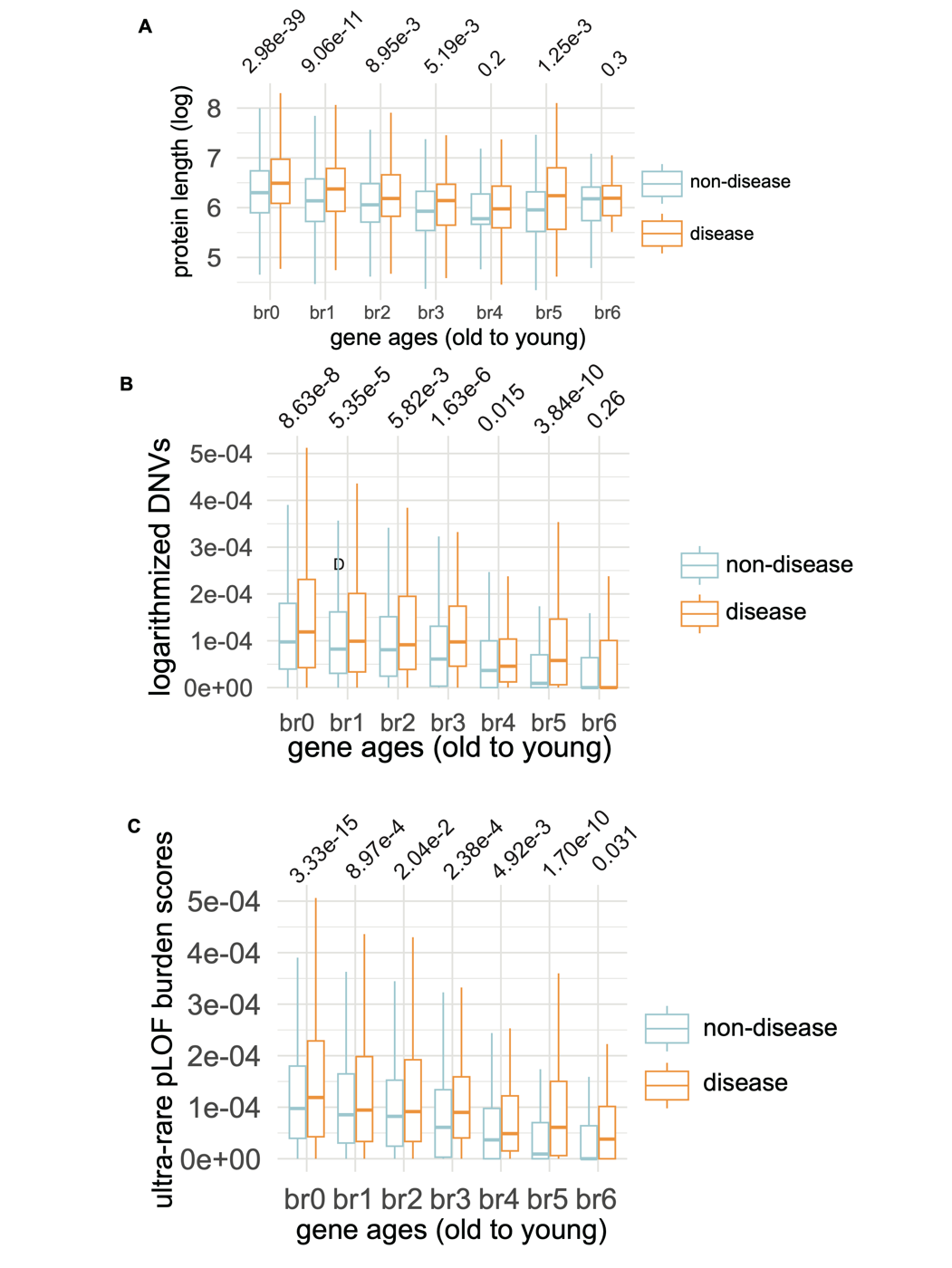


The relationship between multiple features (protein length, burden of DNVs, ultra-rare pLOF burden score) and seven gene age groups (phylostrata). (*A*) The comparison of protein lengths across gene ages between disease genes and non-disease genes. (*B*) The comparison of DNVs burdens across gene ages between disease genes and non-disease genes. (*C*) The comparison of ultra-rare pLOF burden scores across gene ages between disease genes and non-disease genes.

Supplemental_Fig_S4


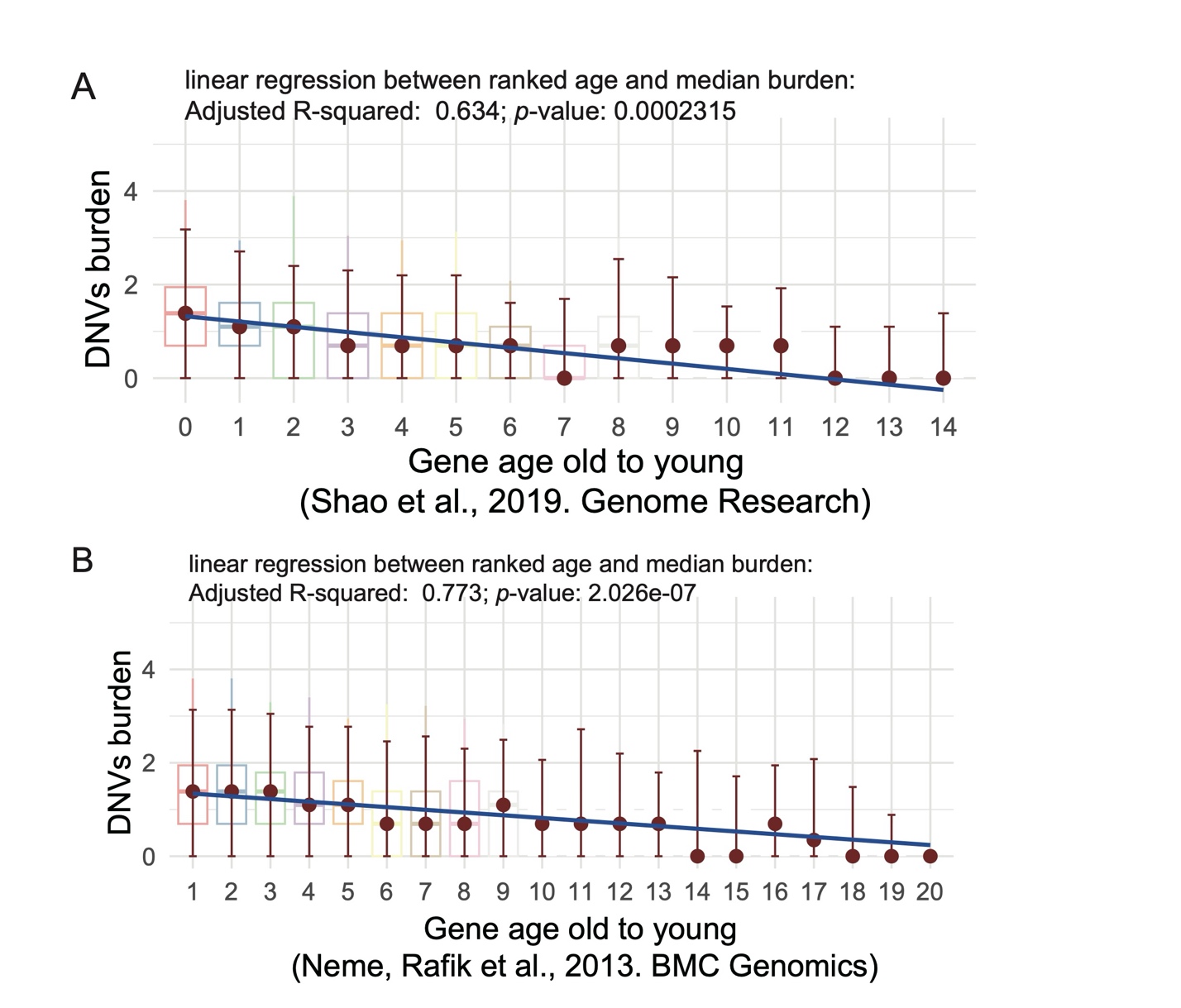


The relationship between two types of gene age dating and the DNVs burden. (*A*)The relationship between gene-wise DNVs burden from 68,404 individuals (Zhao et al. 2020) and the previously reported synteny-based gene age (Shao et al. 2019). (*B*) The relationship between gene-wise DNVs burden from 68,404 individuals (Zhao et al. 2020)and gene-family based gene age (Neme and Tautz 2013). Note: the linear models are between median values and ranked ages. The significance *p* values are shown above age groups (the one-tail Wilcoxon rank sum test with continuity correction).

Supplemental_Fig_S5


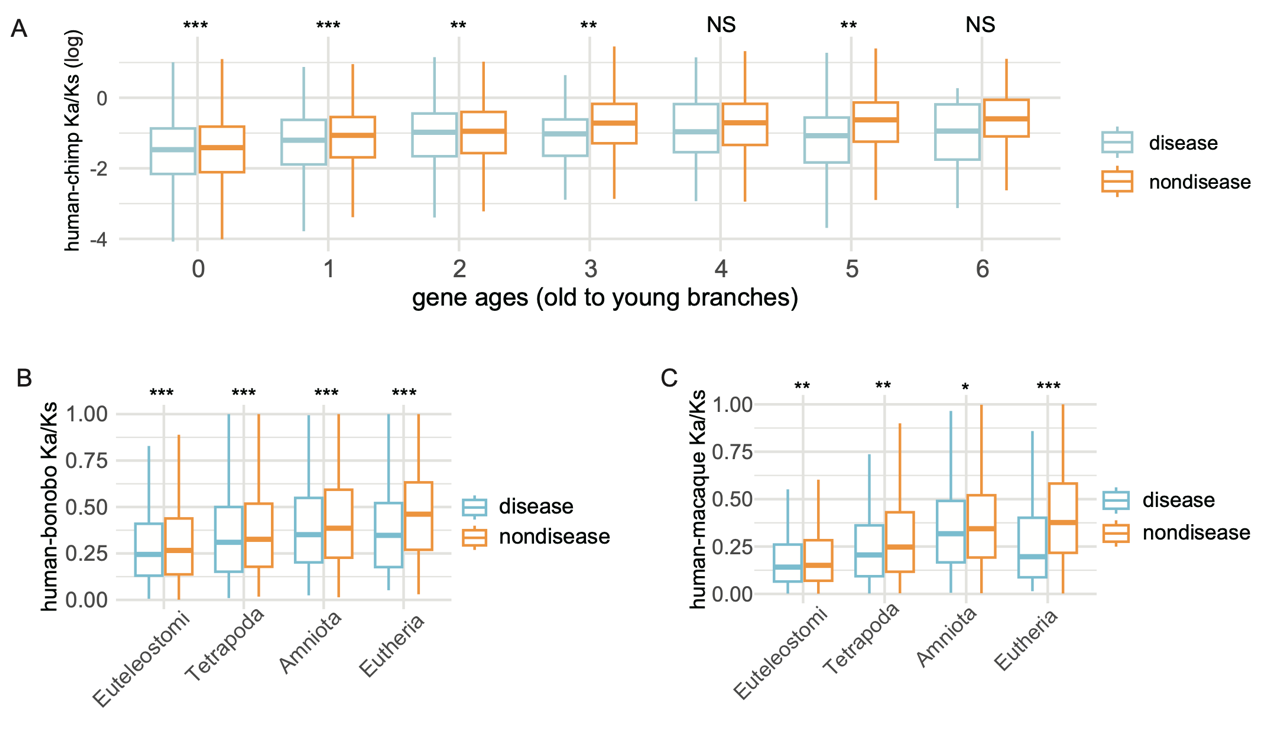


The pairwise Ka/Ks ratios from the Ensembl database based on the Maximum Likelihood estimation for “one-to-one” orthologs between human and other species. (*A*) The pairwise Ka/Ks ratios between human and chimpanzee across seven age groups. (*B*) The pairwise Ka/Ks ratios between human and bonobo across four age groups. (*C*) The pairwise Ka/Ks ratios between human and macaque across four age groups. Only genes under purifying selection are visualized (Ka/Ks < 1). Note: significance levels are based on the Wilcoxon rank sum test comparing disease genes and non-disease genes (one tail test). ”*”, ”**”, “***” indicate p < 0.05, < 0.01, < 0.001, respectively.

Supplemental_Fig_S6


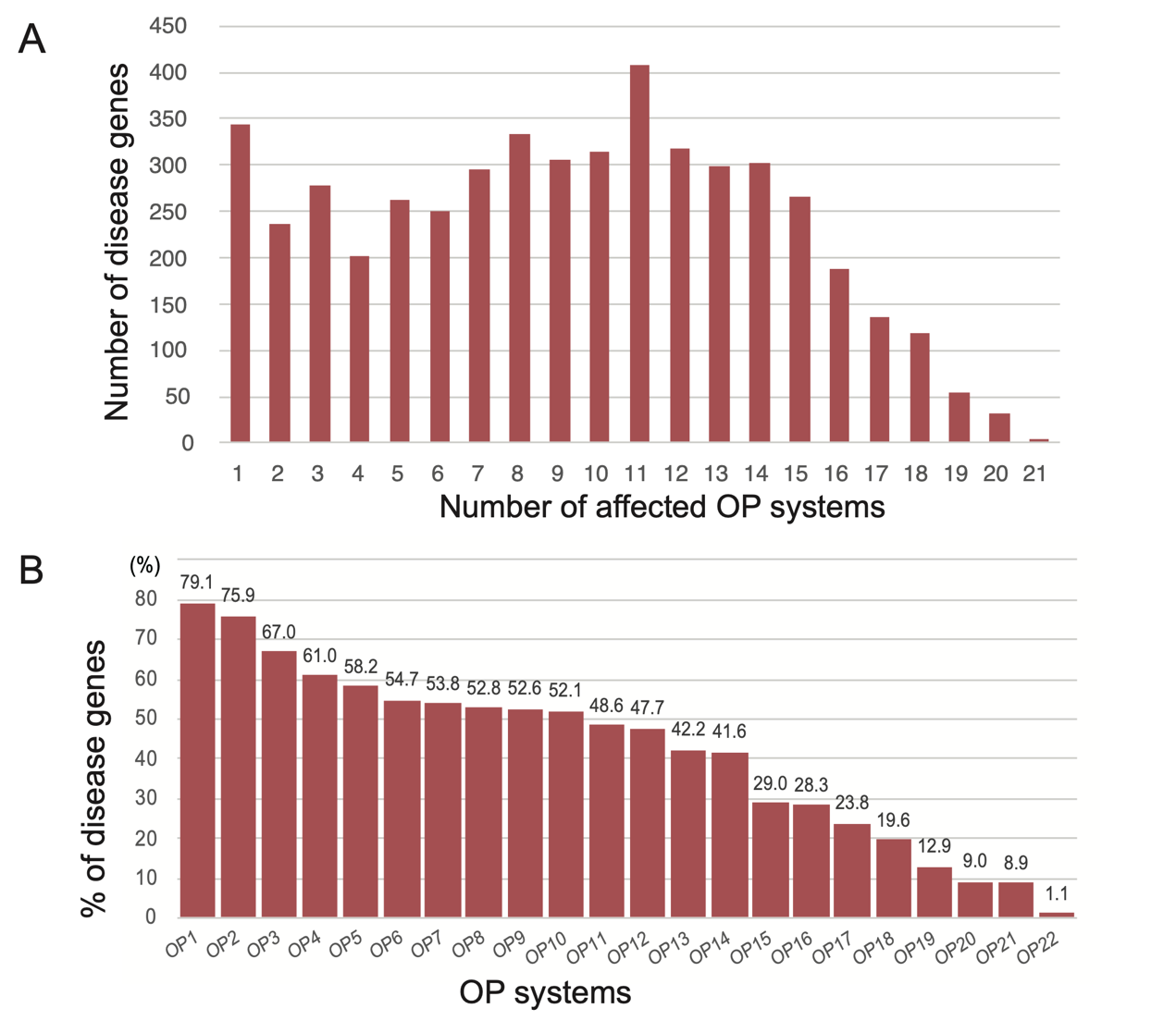


The numbers of disease genes affecting single disease system (OP count = 1) and multiple OP systems (two or more OPs). (*A*) The distribution disease gene counts along the OP numbers. The horizontal axis is based on the numbers of disease systems that a gene defect could impact, and the vertical axis is the number of disease genes. (*B*) The percentage of disease genes for each disease system (OP1 to OP22). The percentage is based on the number of disease genes for certain OP out of all disease genes. The definition of OP systems is consistent with Fig. 1B.

Supplemental_Fig_S7


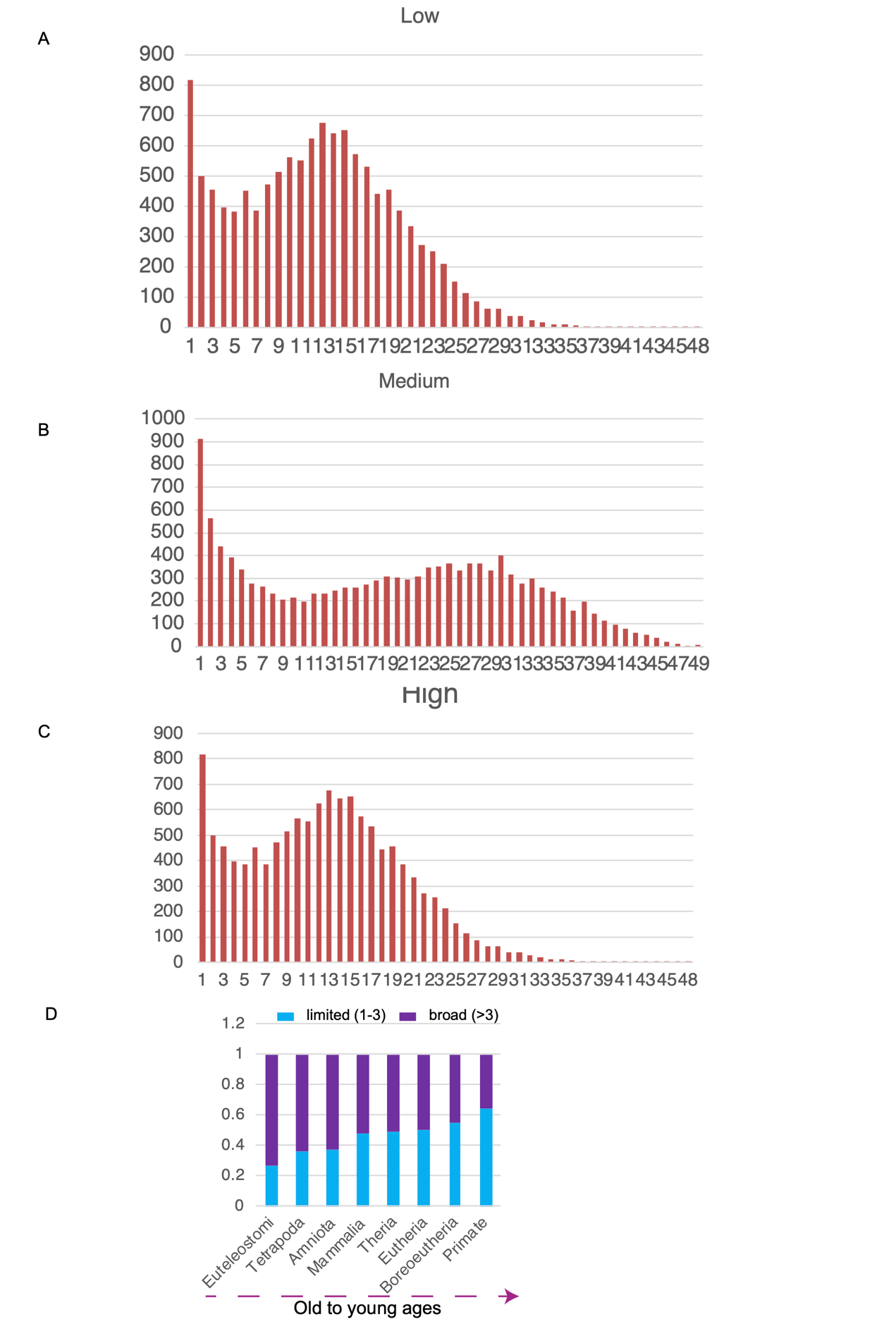


The distribution of gene expression breadth (the number of tissues) with RNASEQ data (The Human Protein Atlas, or HPA, normal tissues). (*A*) The distribution of counts of normal tissues with low levels of gene expression (based on HPA annotation). (*B*) The distribution of counts of normal tissues with medium levels of gene expression (based on HPA annotation). (*C*) The distribution of counts of normal tissues with high levels of gene expression (HPA annotation). (*D*) The distribution percentages of genes expressed in multiple tissues (>3) compared to those expressed in a limited number of tissues (<=3) across different evolutionary ages (only “HIGH” expression genes based on HPA are used).

Supplemental_Table_S1. The gene age information for 19,665 protein-coding genes from autosomes, X, and Y chromosomes.

Supplemental_Table_S2. HPO organ abnormality and genes.

Supplemental_Table_S3. Disease genes states (1 yes; 0 no) and their predictors.

Supplemental_Table_S4. Logistic regression and the model comparison for binary states (“1”, disease genes; “0”, non-disease genes) of all genes.

Supplemental_Table_S5. Burden of deleterious variants between new genes and parental gene.

Supplemental_Table_S6. Ka/Ks for species pairs of human-chimpanzee, human-bonobo, human-macaque.

Supplemental_Table_S7. The disease gene emergence rate, which is the percentage of disease genes for each age per millions of years, r.

Supplemental_Table_S8. Gene expression breadth information based on HPA annotation and the number of affected OP systems for disease genes.

Supplemental_Table_S9. The median values of OP numbers (Figure 2c), phenotype enrichment index (PEI, Figure 2e, see the formula in M&M).

Supplemental_Table_S10. Chromosome Distribution of Disease Genes.

Supplemental_Table_S11. Reproductive system related disease genes.

Supplemental_Table_S12. Male Reproductive system related disease genes and evolutionary strata distribution.
